# Supplementary material for: Exploring relationships between drought and epidemic cholera in Africa using generalised linear models
Source: BMC Infect Dis. 2021 Nov 22;21:1177. doi: 10.1186/s12879-021-06856-4 (PMC8609751; doi:10.1186/s12879-021-06856-4)
Supplement: Supplementary file 1 — Additional file 1. Additional details about the data and models used here. [file 12879_2021_6856_MOESM1_ESM.pdf]

Additional File 1

Exploring the relationships between drought and epidemic cholera in Africa using generalised linear models

Gina E C Charnley, Ilan Kelman, Nathan Green, Wes Hinsley, Katy A M Gaythorpe, Kris A Murray

Information 1. Sensitivity analysis for the assumption that years where no cholera data was reported in the dataset, the outcome variable was set to zero.
Here, three alternative assumptions for the cholera outbreak variable were tested:

Alternative 1:
Removing all the rows with missing cholera data and only using reported 1 and 0s.
Only drought came out as an important predictor in the covariate selection process from 373 data points.

Alternative 2:
Setting all outbreaks to 0.
All variables were removed in the univariate analysis as they did not meet the threshold of $p<0.1$.

Alternative 3:
Setting all outbreaks to 1.
All variables were removed in the univariate analysis as they did not meet the threshold of $p<0.1$.

Table S1. Studies linking cholera outbreaks to risk factors that were used to establish the prior hypothesis for the initial nineteen covariates that were selected as potentially influential factors in cholera outbreak occurrence in Africa. This is additional to those referenced in the manuscript.

Source	Covariate
Nsagha DS, Atashili J, Fon PN, Tanue EA, Ayima CW, Kibu OD. Assessing the risk factors of cholera epidemic in the Buea Health District of Cameroon. BMC Public Health. 2015 Dec;15(1):1-7.	Hygiene and water
Labite, H., Lunani, I., van der Steen, P., Vairavamoorthy, K., Drechsel, P. and Lens, P., 2010. Quantitative Microbial Risk Analysis to evaluate health effects of interventions in the urban water system of Accra, Ghana. *Journal of water and health*, *8*(3), pp.417-430.	Sanitation and water
Alsan MM, Westerhaus M, Herce M, Nakashima K, Farmer PE. Poverty, global health, and infectious disease: lessons from Haiti and Rwanda. Infectious Disease Clinics. 2011 Sep 1;25(3):611-22.	Poverty, health expenditure and development
Talavera A, Perez EM. Is cholera disease associated with poverty?. The Journal of Infection in Developing Countries. 2009 Jul 1;3(06):408-11.	Poverty
Gidado S, Awosanya E, Haladu S, Ayanleke HB, Idris S, Mamuda I, Mohammed A, Michael CA, Waziri NE, Nguku P. Cholera outbreak in a naïve rural community in Northern Nigeria: the importance of hand washing with soap, September 2010. The Pan African Medical Journal. 2018;30.	Handwashing
Aggrey-Korsah E, Oppong J. Researching urban slum health in Nima, a slum in Accra. InSpatial inequalities 2013 (pp. 109-124). Springer, Dordrecht.	Informal settlement
Penrose, K., de Castro, M.C., Werema, J. and Ryan, E.T., 2010. Informal urban settlements and cholera risk in Dar es Salaam, Tanzania. *PLoS Negl Trop Dis*, *4*(3), p.e631.	Informal setttlement, population density
Ververs M, Narra R. Treating cholera in severely malnourished children in the Horn of Africa and Yemen. The Lancet. 2017 Oct 28;390(10106):1945-6.	Malnourishment
Osei FB, Duker AA. Spatial and demographic patterns of cholera in Ashanti region-Ghana. International Journal of Health Geographics. 2008 Dec;7(1):1-0.	Population, total and density

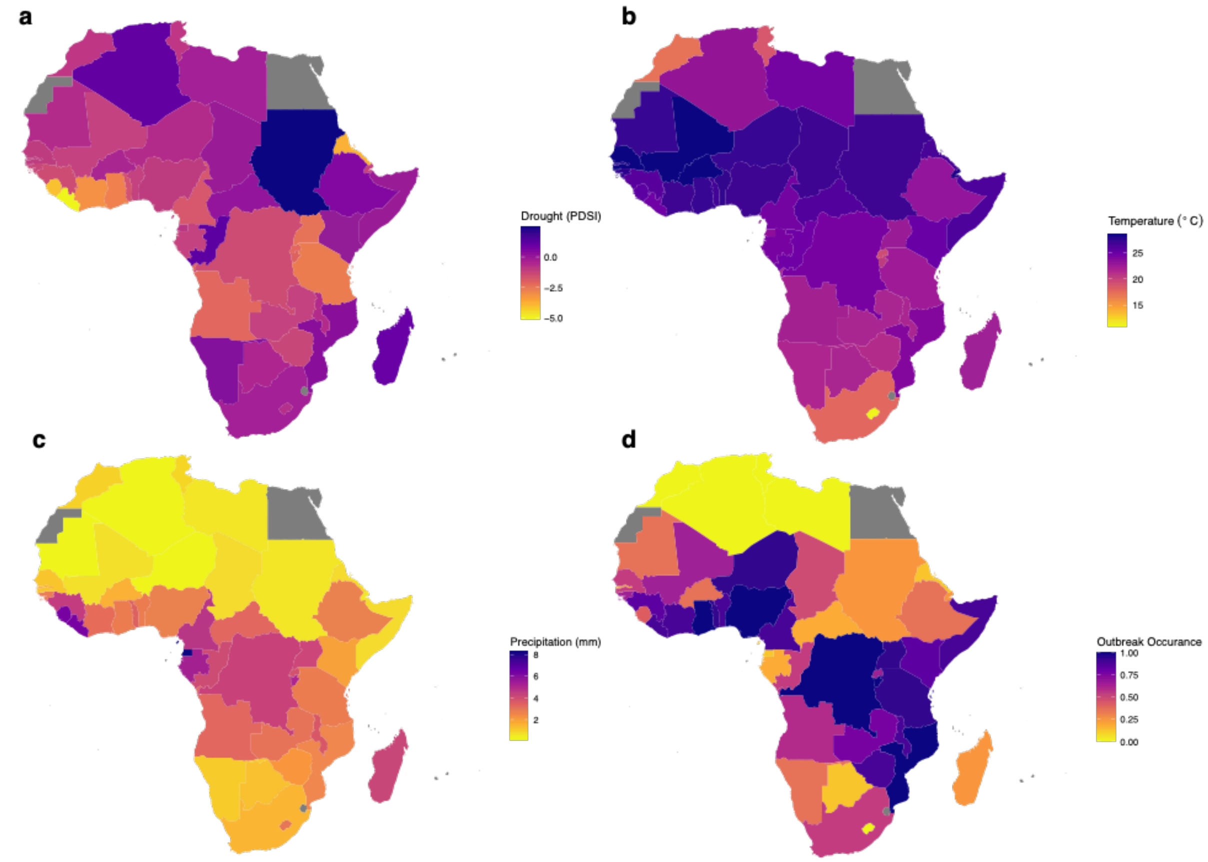

Fig. S1. National average for the instrumental period (2000-2016) for **a**, mean drought data, **b**, average temperature data and **c**, average precipitation data and **d**, mean cholera outbreak occurrence.

The map is our own work and the shapefiles are taken from: https://thematicmapping.org/downloads/world_borders.php, under CC-BY SA, allowing them to be shared and adapted.

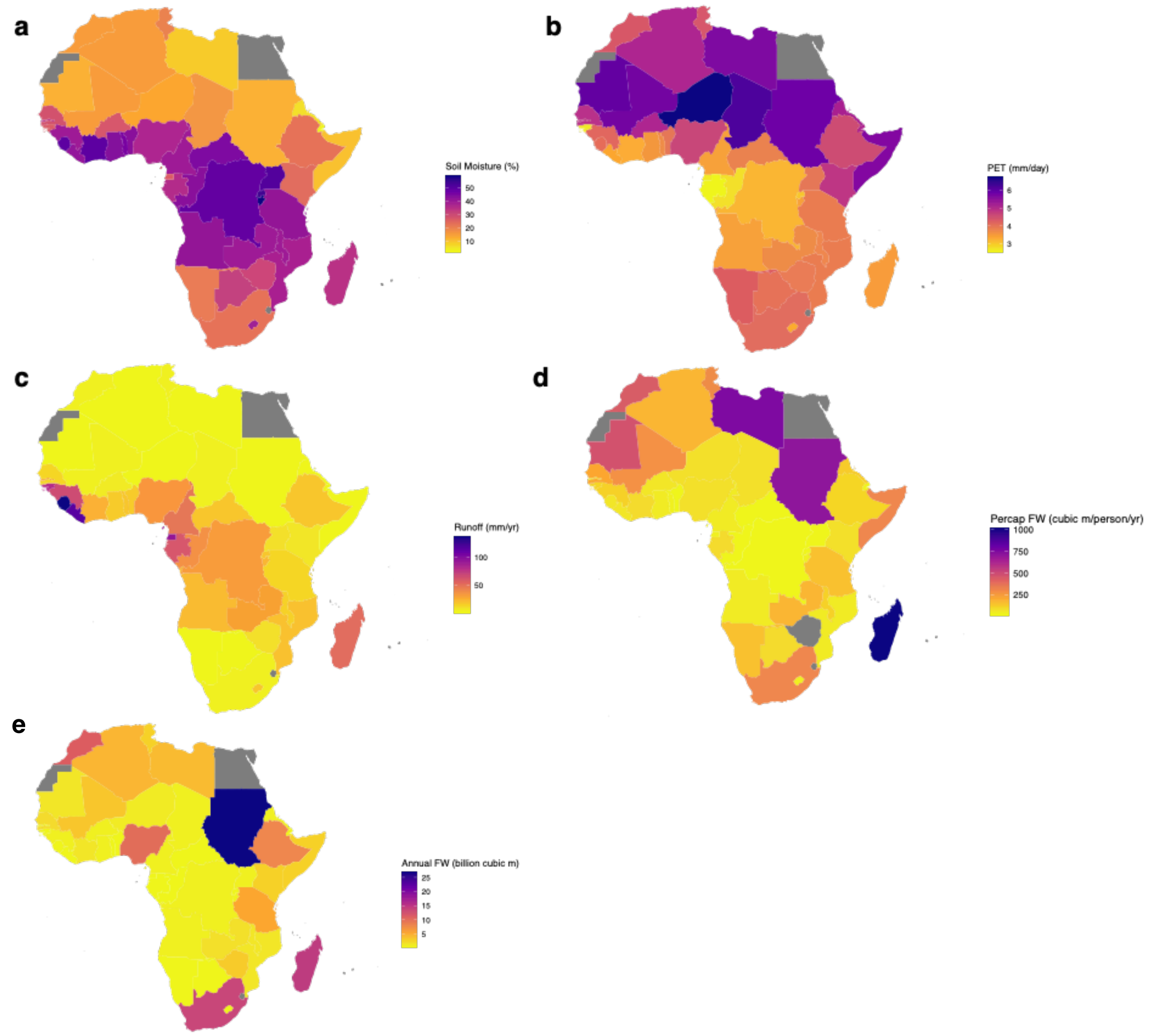

Fig. S2. National average for the instrumental period (2000-2016) for agricultural drought indices; **a**, soil moisture (%) and **b**, potential evapotranspiration (mm/day) and hydrological drought indices; **c**, runoff (mm/year), **d**, per capita freshwater withdrawal (m^3/person/yr) and **e**, annual freshwater withdrawal (billion m^3).
The map is our own work and the shapefiles are taken from: https://thematicmapping.org/downloads/world_borders.php, under CC-BY SA, allowing them to be shared and adapted.

Information 2.

Palmer Drought Severity Index - The calculation of PDSI relies on a water balance-based two-bucket system, where there is a surface layer with a storage capacity of 1 in and an underlying layer with a storage capacity of -1 (Jacobi *et al*., 2013). It was first developed by Palmer (1965) and uses temperature and potential evapotranspiration (PET) data, capturing the basic effects of climate change through PET changes (Dai & NCAR, 2019). The PDSI was used to quantifying long-term drought and was taken as a national average from the annual admin one data.

Sources: Jacobi, J., Perrone, D., Duncan, L.L. and Hornberger, G., 2013. A tool for calculating the Palmer drought indices. Water Resources Research, 49(9), pp.6086-6089.

Palmer, W.C., 1965. Meteorological drought (Vol. 30). US Department of Commerce, Weather Bureau.

Dai, A. and National Centre for Atmospheric Research. The Climate Data Guide: Palmer Drought Severity Index (PDSI). [On-line]. Available from: https://climatedataguide.ucar.edu/climate-data/palmer-drought-severity-index-pdsi (accessed 17 August 2020).

Soil moisture - The dataset provides estimates of soil moisture from satellite sensors over the globe. It is based on the European Space Agency's Climate Change Initiative soil moisture version 03.3. Data was provided as a netCDF file for each month from 2000-2016 on a regular latitude/longitude grid at 0.25x0.25 resolution on a monthly temporal resolution. This data was processed by transforming the co-ordinates to each country (by creating a function using the R packages sp and rworldmap) and creating a national annual mean. This provided surface soil moisture as a percentage content of liquid water in a surface soil layer of 2-5cm depth expressed as the percentage of total saturation.

Source: Copernicus, 2018. Soil moisture gridded data from 1978 to present. [On-line]. European Centre for Medium-Range Weather Forecasts. Available from: https://cds.climate.copernicus.eu/cdsapp#!/dataset/satellite-soil-moisture?tab=form (accessed 26 October 2020).

Potential Evapotranspiration - This data was taken from the National Centre for Atmospheric Research (NCAR) Climate data guide and comes from the Climate Research Unit Timeseries (CRU TS) series of datasets, specifically CRU TS4.0. The data contains several climate fields including precipitation, temperature and cloud cover, which is used to compute variables including evapotranspiration in mm per day. The data covers all land surface and spans from 1901-2015, although data here was only extracted for 2001-2015, as this best captures the instrumental period of this research. The data is gridded to 0.5x0.5-degree resolution based on 4000 individual weather station data sources; this requires homogenisation which can introduce limitations as the data will not be strictly homogenous. The data is provided in two netCDF format cells (2001-2010 and 2011-2015), which was then extracted into a .csv file as a national average for each year.

Source: NCAR, 2015. CRU TS GRIDDED PRECIPITATION AND OTHER METEOROLOGICAL VARIABLES SINCE 1901. [On-line]. Available from: https://climatedataguide.ucar.edu/climate-data/cru-ts-gridded-precipitation-and-other-meteorological-variables-1901 (accessed 26 October 2020).

Runoff - Observed river discharge information and a climate driven Water Balance Model was combined in order to develop composite runoff fields which are consistent with observed discharges. The aim of the methods used is to provide a best estimate of terrestrial runoff over large domains in mm per year, which is needed for this study. The composite fields use data from a gridded river network at 30-minute spatial resolution to represent the riverine flow pathways and to link the continental land mass to oceans through river channels. This used selected gauging stations from the Global Runoff Data Centre (GRDC). Inter-station discharge and runoff were calculated to compare observed runoff with outputs from water balance model simulations. Correction coefficients based on the ratio of observed and simulated runoff for inter-station areas were calculated and applied against simulated runoff to create composite runoff fields. The GRDC also provided data for each station as individual .txt files but once processed the files were missing large amounts of data and therefore the composite fields were instead used, given as .grd files for each month.
Source: UNH/GRDC, 2000. UNH/GRCD Composite Runoff Fields V1.0. [On-line]. Available from: https://www.compositerunoff.sr.unh.edu (accessed 26 October 2020).

Annual freshwater withdrawal - Is provided as a .csv file containing county, year and annual freshwater withdrawals in billion m^3 from 1962-2016. All data outside the instrumental period (2000-2016) was removed and a national average taken. Annual freshwater withdrawals refer to total water withdrawals, not counting evaporation losses from storage in basins. Withdrawals also include water from desalination plants in countries where they are a significant source. Withdrawal for agriculture and industry are total withdrawals for irrigation and livestock production and for direct industrial use. Domestic uses include drinking water, municipal use or supply and public services. The source of this data is the Food and Agriculture Organization's AQUASTAT data. Data is collected intermittently and subject to variations in collection and estimation methods which may hide significant variations in water availability within countries. Data for smaller countries or in arid and semi-arid areas are less reliable than those for large countries and countries with greater rainfall.

Per capita freshwater withdrawal - The source of the per capita data was also AQUASTAT and presented in the same way. The dataset time span was 1960-2015 and is calculated from the annual quantity of water withdrawn for agricultural, industrial and municipal purposes. It can include water from primary and secondary resources as well as from over-abstraction of renewable groundwater, fossil groundwater, agricultural drainage water, treated wastewater and desalinated water. It does not include in-stream use which typically has very low net consumption rates such as recreation, navigation, hydropower and inland capture fisheries.
Source: Ritchie, H., 2017. Water Use and Stress. [On-line]. Our World In Data. Available from: https://ourworldindata.org/water-use-stress (accessed 26 October 2020).

Table S2. Correlation matrix for the nineteen covariates included in the selection criteria for consideration in the final model.

		1	2	3	4	5	6	7	8	9	10	11	12	13	14	15	16	17	18	19
1	**Mean Drought**	1																		
2	**PET**	0.51	1																	
3	**Soil Moisture**	-0.66	-0.83	1																
4	**Runoff**	-0.7	-0.67	0.72	1															
5	**Withdrawal-Annual**	0.48	0.29	-0.5	-0.49	1														
6	**Withdrawal-PerCapita**	0.54	0.34	-0.64	-0.39	0.79	1													
7	**Avg. Temperature**	-0.05	0.49	-0.16	0.08	-0.31	-0.2	1												
8	**Avg. Precipitation**	-0.68	-0.85	0.92	0.91	-0.45	-0.49	-0.12	1											
9	**Population (log)**	0.26	0.41	-0.27	-0.53	0.72	0.27	0	-0.38	1										
10	**Poverty Headcount**	-0.15	-0.05	0.45	0.41	-0.23	-0.26	0.28	0.44	0.06	1									
11	**Informal Settlement**	-0.07	0.19	0.23	0.33	-0.25	-0.2	0.57	0.27	0.08	0.9	1								
12	**Malnourishment**	-0.19	-0.28	0.55	0.48	-0.36	-0.31	0.14	0.57	-0.1	0.83	0.78	1							
13	**Sanitation**	0.1	-0.07	-0.32	-0.44	0.3	0.22	-0.59	-0.39	0.01	-0.89	-0.95	-0.79	1						
14	**Handwashing**	0.37	0.12	-0.55	-0.57	0.54	0.58	-0.49	-0.56	0.15	-0.79	-0.81	-0.65	0.8	1					
15	**GDP**	0.2	0.14	-0.36	-0.49	0.74	0.32	-0.37	-0.41	0.62	-0.56	-0.59	-0.66	0.65	0.59	1				
16	**Drinking Water**	-0.04	-0.22	-0.21	-0.17	0.11	0.15	-0.46	-0.2	-0.28	-0.9	-0.96	-0.8	0.9	0.72	0.49	1			
17	**HDI**	0.08	-0.27	-0.22	-0.27	0.35	0.32	-0.64	-0.21	-0.04	-0.88	-0.96	-0.71	0.92	0.84	0.62	0.92	1		
18	**Health Expenditure**	-0.49	-0.23	0.28	0.4	-0.31	-0.23	-0.31	0.32	-0.35	0.18	0.03	0.13	-0.02	-0.18	-0.26	0.04	-0.06	1	
19	**Population Density**	-0.47	-0.41	0.6	0.14	-0.22	-0.53	-0.29	0.38	0.08	0.16	-0.06	0.06	0.13	-0.32	0.06	0	-0.01	0.32	1

a

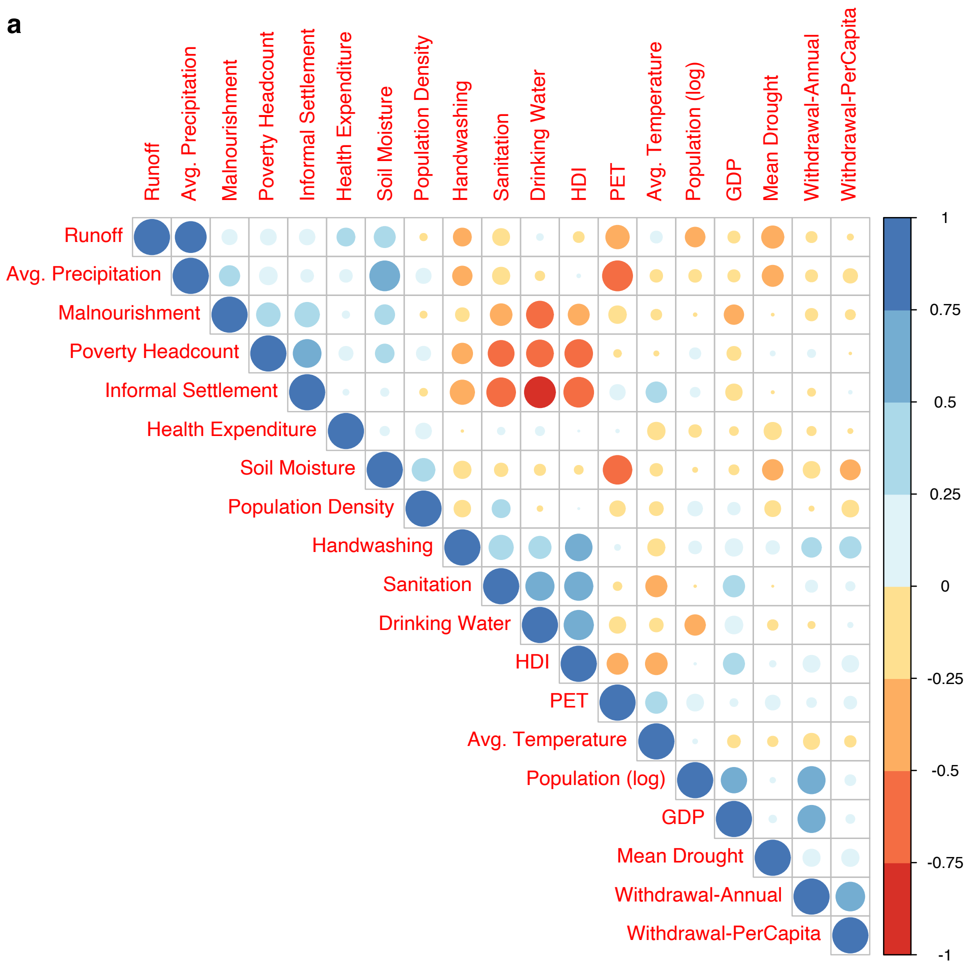

b

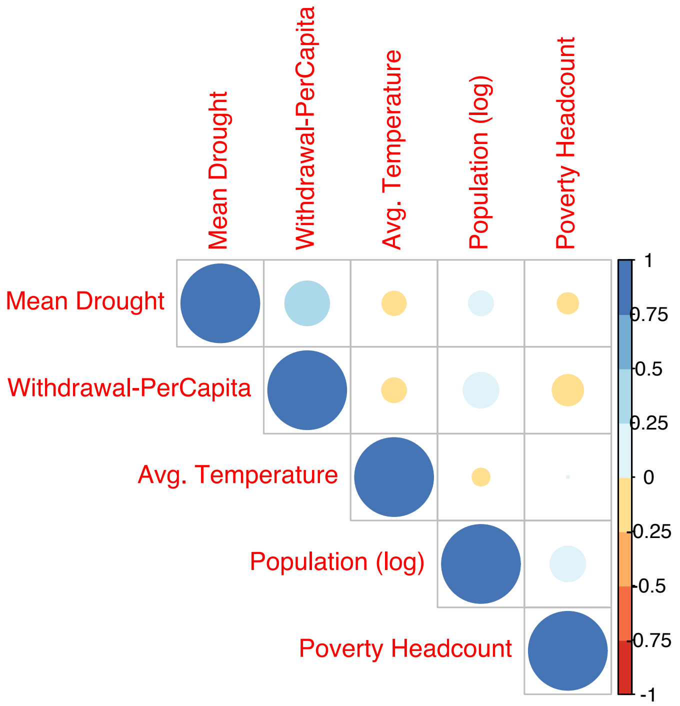

Fig. S3. Correlation plots of the **a**, nineteen covariates included in the covariate selection criteria and **b**, the five covariates included in the best-fit model

Information 3.
Projected temperature data were available through WorldClim, which gives projections for 2050 and 2070, under the four Representative Concentration Pathways (RCP) emissions scenarios. RCP2.6 requires emissions to start declining by 2020 and reach net zero by 2100, RCP4.5 requires emissions to peak in 2040, RCP6.0 peaks at around 2080 and RCP8.5 assumes emission will continue to rise throughout the century. These four scenarios are projected to have mean global warming by 2081-2100 of 1°C, 1.8°C, 2.2°C and 3.7°C, respectively (IPCC, 2014). RCP2.6 was not used in the projections, as this requires CO_2 emissions to peak in 2020, a goal which has not been reached (NOAA, 2020). The data was presented as a monthly average at the admin 1 level, which was then transformed into a national yearly mean. Summary plots of the projected temperature data are shown in Appendix 7 Figure 3.

Projected PDSI data was difficult to obtain, and previous modelling studies have found spatial heterogeneity, making projecting drought across the continent challenging. There is also disagreement over drought changes in Africa under climate change and how populations will adapt to alterations in water scarcity (Ahmadalipour *et al.*, 2019; Calow *et al.*, 2010; Haile *et al.*, 2020; Shanahan *et al.*, 2009; Touchan *et al.*, 2008; Verschuren *et al.*, 2000). One of the main difficulties in calculating projected PDSI and discrepancies in results is the algorithm used to calculate PET (Tian-Jun and Tao, 2013). PET algorithms do not account for changes in vegetation cover expected due to elevated CO_2, making the "warming leads to drying" narrative flawed and over-simplistic, causing overestimations (Yang *et al.*, 2020). Several studies which have taken a more critical approach of the methodology found that drought did not significantly change over long periods and changes are likely to be seen on finer spatial scales (Sheffield *et al.*, 2012; Yang *et al.*, 2020). These findings are consistent with tree-ring (Touchan *et al.*, 2008), lake sediment records (Verschuren *et al.*, 2000) and the PDSI dataset used here. This suggests that droughts are neither more severe nor longer now than historically. Despite the large number of projected drought studies, extracting these results and using them here is challenging, as they are on different spatial scales, using different methodology and data. There are also issues with the projected indices used, as these often vary and are potentially not comparable. To account for this, three scenarios were created from historical PDSI data using univariate linear regression models for drought and year. This method used the coefficients for each country to project the future drought data, with scenario 3 continually plotting the coefficient increase until 2070 (or it reached -4 or 4, the extremes of the PDSI scale), scenario 1 accepting the above hypotheses that PDSI is an overestimation and drought will not change over the projected period and scenario 2 as a median value. Appendix 9 Figure 6 shows the linear regression coefficients for each country during the instrumental period (2000-2016).

Projected population data was based on the United Nation's World Population Prospectus (2019). The projections are based on available data on population size, levels of fertility, mortality and international migration. Data is from censuses, registration of births and deaths, demographic and health surveys, official statistics and population registers. More recently, the data has taken into account refugee statistics, prevalence of HIV and antiretroviral coverage, infant and under five morality and migration flows. The projections use the cohort component method using a variety of demographic assumptions concerning fertility,

mortality and migration. This takes into account the past experiences of the country and reflects uncertainty and other countries in similar conditions. The medium variant projection corresponds to the median of several trajectories of each demographic component derived using the probabilistic model of the historical variability over time. Prediction intervals represent the spread in the distribution of outcomes across the projected trajectories and thus provide and assessment of the uncertainty inherent in the medium variant projection. Therefore, only the medium variant projection was included in the model and not as an offset.

Projected poverty headcount at <$1.90 a day was based on Sustainable Development Goal (SDG) 1 (UN, 2015), which states that by 2030, extreme poverty (<$1.25/day) will be eliminated and the population living in poverty will be reduced by >50%. This goal is very ambitious and will require significant human and economic resources. Several of the terms within the SDGs are also ambiguous, meaning the aims and roadmap to achieve them are not clear. With regards to poverty, the setting used in the SDGs is slightly lower ($1.25 compared to $1.90) and it is difficult to distinguish the level of poverty within the data; therefore, the projected scenario will mainly align with the second part of the goal, to halve the population in poverty by 2030. Despite their limitations, the SDGs provide a globally recognised pathway to a sustainable future and what institutions and governments should be aiming to achieve, making their use important in scientific research.

Creating projected freshwater withdrawal per capita data for the scenarios also presented challenges, as this is largely down to human behaviour and therefore hard to predict. While SDG6.4 states that by 2030, water use efficiency will be sustainably increased across all sectors. Projected data is not freely available and may not take into account climate change and alterations in societal behaviour, all of which will alter water stress in the future. To maintain sustainable levels of water resources, rates of withdrawal need to be lower than replenishment. Renewable resources come from internal river flows and groundwater from rainfall. To understand the national historical water security, data was plotted for both freshwater withdrawal and freshwater resources (Ritchie, 2017) (Appendix 11 Figure 8). The figure helps to illustrate that most countries in Africa have both low resources and use, making the likely use of freshwater sustainable in most countries. Exceptions to this are Gabon, Republic of Congo and Liberia which have comparatively high resources and low withdrawal as well as Madagascar, Libya, Sudan, Mauritania and Morocco which have comparatively low resources and high use. Given the relationship between cholera outbreaks and water withdrawal, most countries in Africa could increase their freshwater withdrawal, except for Madagascar, Libya, Sudan, Mauritania and Morocco which should reduce their use, to improve sustainability.

Sources: Intergovernmental Panel on Climate Change, 2014. Climate Change 2014 Synthesis Report Fifth Assessment Report Future Climate Changes, Risks and Impacts. [On-line]. Available from: https://ar5-syr.ipcc.ch/topic_futurechanges.php (accessed 30 October 2020).
NOAA, 2020. Trends in Atmospheric Carbon Dioxide. [On-line]. Available from: https://www.esrl.noaa.gov/gmd/ccgg/trends/ (accessed 30 October 2020).
Ahmadalipour, A., Moradkhani, H., Castelletti, A. and Magliocca, N., 2019. Future drought risk in Africa: Integrating vulnerability, climate change, and population growth. Science of the Total Environment, 662, pp.672-686.

Calow, R.C., MacDonald, A.M., Nicol, A.L. and Robins, N.S., 2010. Ground water security and drought in Africa: linking availability, access, and demand. Groundwater, 48(2), pp.246-256.
Haile, G.G., Tang, Q., Hosseini‐Moghari, S.M., Liu, X., Gebremicael, T.G., Leng, G., Kebede, A., Xu, X. and Yun, X., 2020. Projected impacts of climate change on drought patterns over East Africa. Earth's Future, 8(7), p.e2020EF001502.
Shanahan, T.M., Overpeck, J.T., Anchukaitis, K.J., Beck, J.W., Cole, J.E., Dettman, D.L., Peck, J.A., Scholz, C.A. and King, J.W., 2009. Atlantic forcing of persistent drought in West Africa. Science, 324(5925), pp.377-380.
Touchan, R., Anchukaitis, K.J., Meko, D.M., Attalah, S., Baisan, C. and Aloui, A., 2008. Long term context for recent drought in northwestern Africa. Geophysical Research Letters, 35(13).
Verschuren, D., Laird, K.R. and Cumming, B.F., 2000. Rainfall and drought in equatorial east Africa during the past 1,100 years. Nature, 403(6768), pp.410-414.
Tian-Jun, Z. and Tao, H., 2013. Projected changes of palmer drought severity index under an RCP8. 5 scenario. Atmospheric and Oceanic Science Letters, 6(5), pp.273-278.
Yang Y,, Zhang S., Roderick M. L., McVicar, T. R., Yang D., Liu W. And Li X. 2020. Little change in Palmer Drought Severity Index across global land under warming in climate projections. Hydrology and Earth System Sciences Discussions.
Sheffield, J., Wood, E.F. and Roderick, M.L., 2012. Little change in global drought over the past 60 years. Nature, 491(7424), pp.435-438.
United Nations, 2019. World Population Prospectus 2019. [On-line]. Available from: https://population.un.org/wpp/ (accessed 27 October 2020).
United Nations, 2015. The 17 Goals. [On-line]. United Nations Department for Economic and Social Affairs. Population Dynamics. Available from: https://sdgs.un.org/goals (accessed 27 October 2020).
Ritchie, H., 2017. Water Use and Stress. [On-line]. Our World In Data. Available from: https://ourworldindata.org/water-use-stress (accessed 26 October 2020).

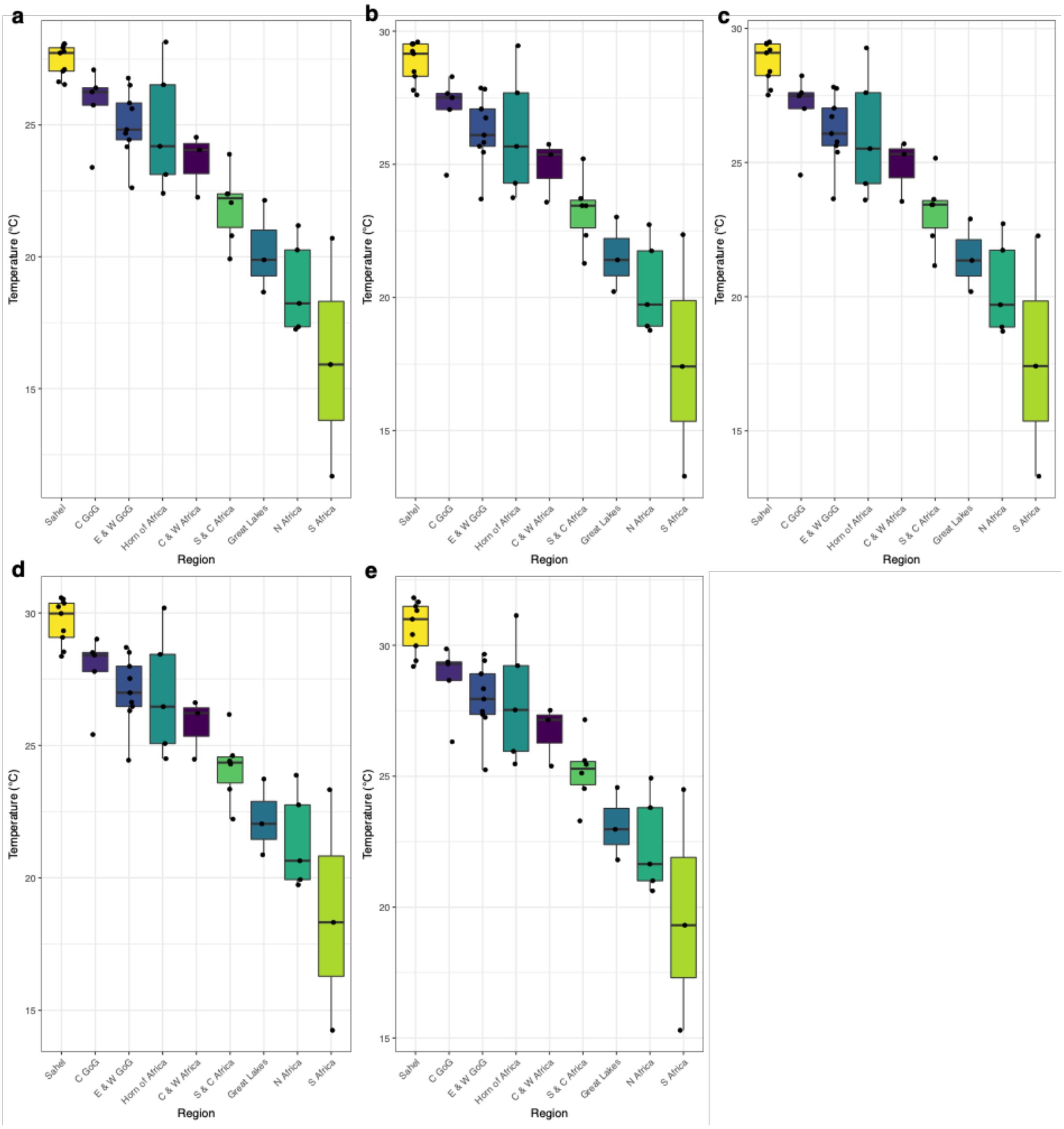

Fig. S4. Summary boxplots with jitters of the projected temperature data from WorldClim for **a**, 2016, **b**, RCP2.6 2050, **c**, RCP2.6 2070, **d,** RCP8.5 2050 and **e**, RCP8.5 2070. C & W Africa: Central and West Africa (Angola, Congo, Democratic Republic of Congo). C GoG: Central Gulf of Guinea (Benin, Côte d'Ivoire, Ghana, Togo, Tanzania). E & W GoG: East and West Gulf of Guinea (Cameroon, Central African Republic, Equatorial Guinea, Gabon. Guinea, Liberia, Nigeria, Sierra Leone, South Sudan). Great Lakes (Burundi, Rwanda, Uganda). Horn of Africa (Djibouti, Eritrea, Ethiopia, Kenya, Somalia). N Africa: Northern Africa (Algeria, Libya, Morocco, Tunisia). S & C Africa: South and Central Africa (Madagascar, Malawi, Mozambique, Namibia, Zambia, Zimbabwe). S Africa: Southern Africa (Botswana, Lesotho, South Africa). Sahel (Burkina Faso, Chad, Gambia, Guinea-Bissau, Mali, Mauritania, Niger, Senegal, Sudan).

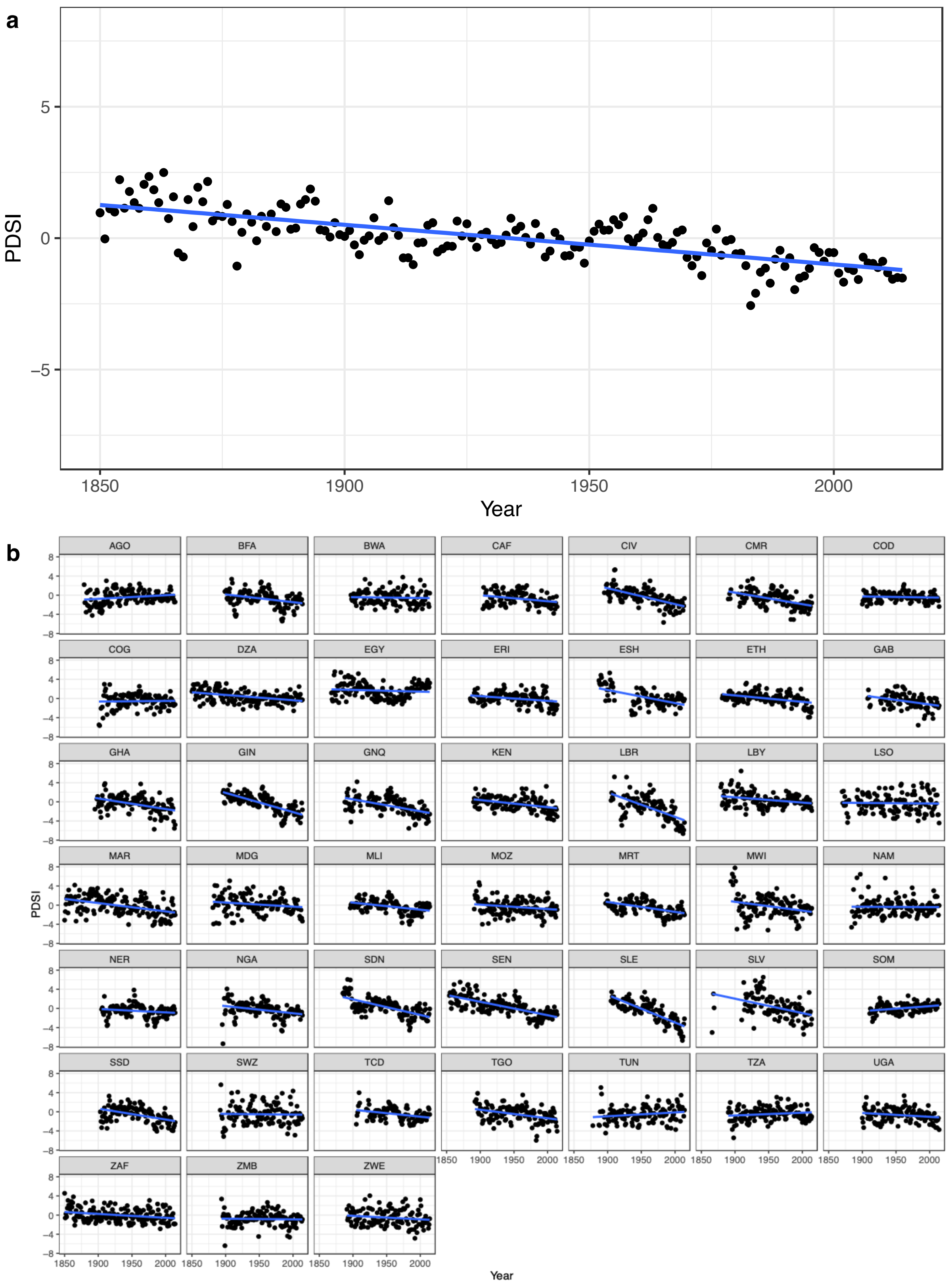

Fig S5. Historical trends in PDSI on **a**, averaged over a continental scale and **b**, averaged over a national scale for the full dataset (1879-2016). Only 8 (BWA, COD, COG, NAM, NER, TZA, ZMB, ZWE) of the 47 countries showed an insignificant cholera trend at p=<0.05.

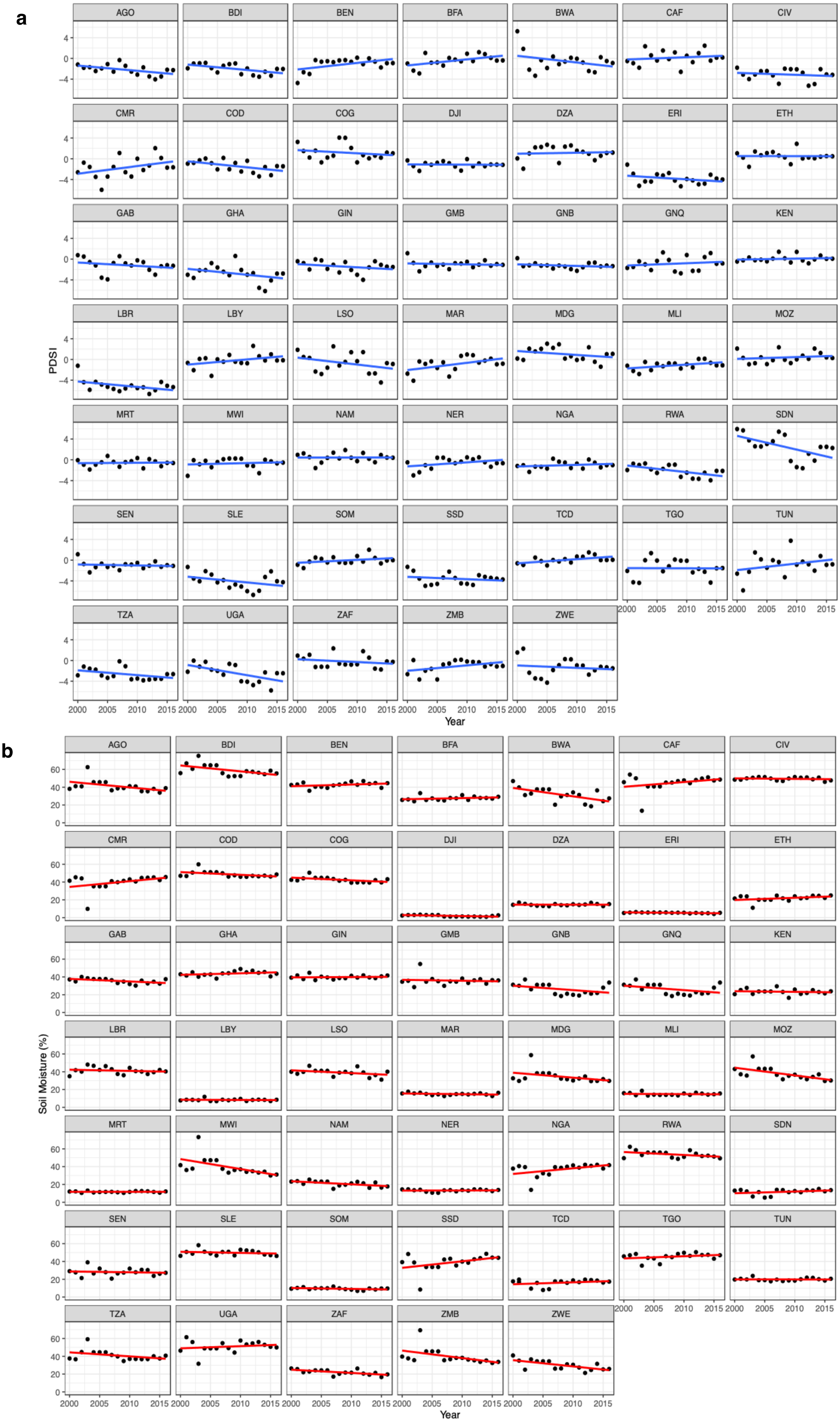

a
AGO BDI BEN BFA BWA CAF CIV
CMR COD COG DJI DZA ERI ETH
GAB GHA GIN GMB GNB GNQ KEN
LBR LBY LSO MAR MDG MLI MOZ
MRT MWI NAM NER NGA RWA SDN
SEN SLE SOM SSD TCD TGO TUN
TZA UGA ZAF ZMB ZWE
PDSI
Year
b
AGO BDI BEN BFA BWA CAF CIV
CMR COD COG DJI DZA ERI ETH
GAB GHA GIN GMB GNB GNQ KEN
LBR LBY LSO MAR MDG MLI MOZ
MRT MWI NAM NER NGA RWA SDN
SEN SLE SOM SSD TCD TGO TUN
TZA UGA ZAF ZMB ZWE
Soil Moisture (%)
Year

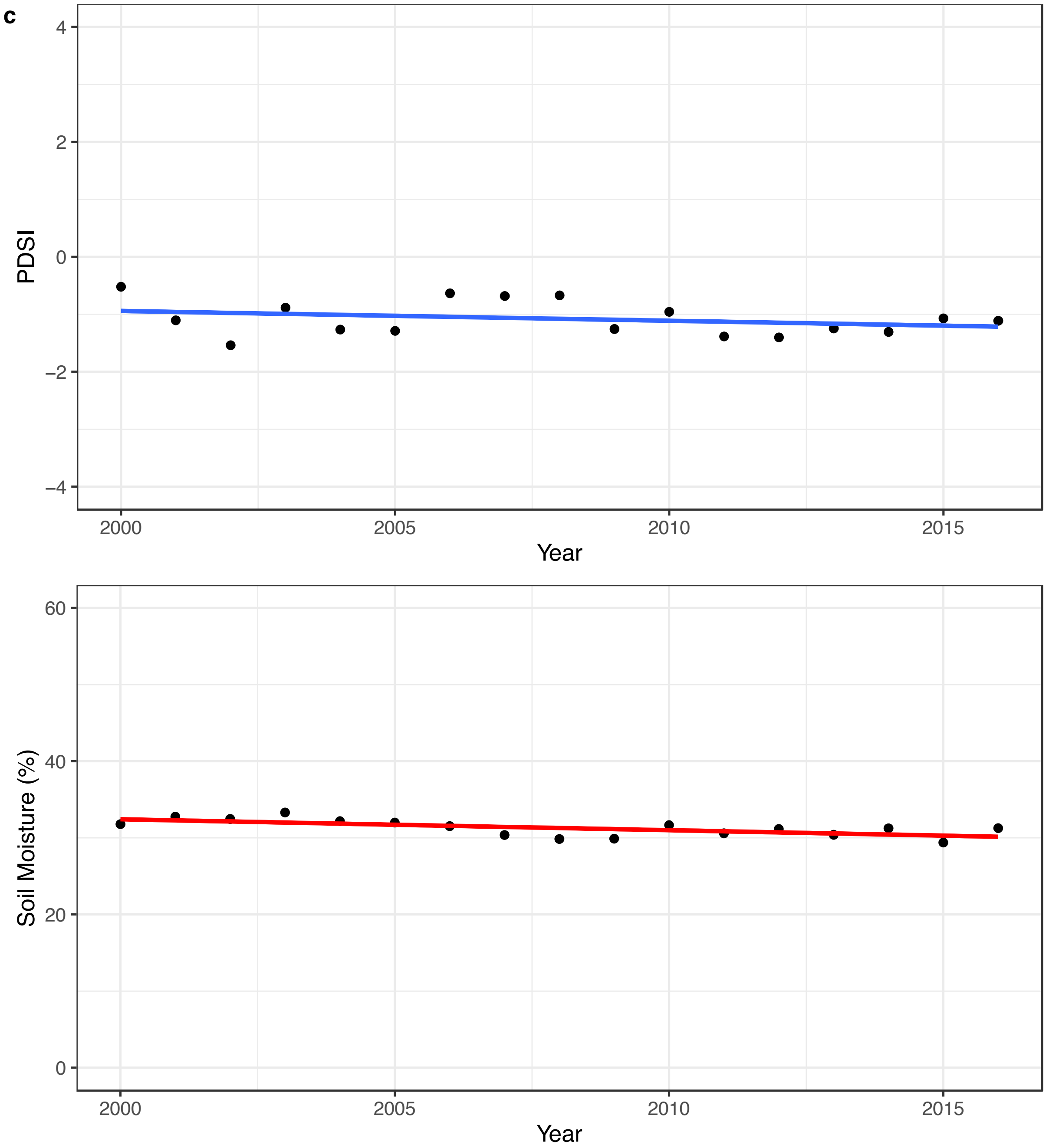

Fig. S6. A comparison of historical trends in **a**, national PDSI, **b**, national soil moisture and **c**, continental averages of PDSI and soil moisture for each year from 2000-2016, the linear relationship between soil moisture and PDSI is significant at $p=7.21x10^{-16}$. We did not compare soil moisture to drought for the full length of the PDSI dataset (1850-2016), as the soil moisture data was only available from 2000.

Table S3. Linear model results for the S3 drought extrapolation projections, Res = Residual

Code	Coeff	P value	SE	R^2	Res. Min	Res. 1Q	Res. Median	Res. 3Q	Res. Max
AGO	0.007584	1.38E-02	0.003	0.0405	-3.4003	-0.9893	-0.1943	1.0217	3.6879
BFA	-0.016124	4.80E-04	0.004	0.1018	-4.3346	-0.7317	0.0433	1.0693	3.4803
BWA	-0.001625	6.84E-01	0.004	0.001437	-2.7141	-1.1711	-0.2378	0.9759	4.3036
CAF	-0.012484	2.20E-03	0.004	0.08274	-2.7737	-0.9622	-0.1477	0.9576	2.8916
CIV	-0.033197	6.41E-15	0.004	0.4091	-4.3856	-0.8907	0.1215	0.7528	4.2415
CMR	-0.02412	1.21E-08	0.004	0.2526	-3.6014	-0.8186	-0.1533	0.992	3.515
COD	-0.002054	4.30E-01	0.003	0.005524	-2.0415	-0.6651	0.01018	0.6305	2.6456
COG	0.001273	7.72E-01	0.004	0.0007691	-5.0383	-0.806	0.1266	0.9635	3.6098
DZA	-0.011053	1.71E-08	0.002	0.1777	-2.7972	-0.82673	0.07252	0.70509	2.95153
ERI	-0.009764	1.18E-03	0.082	0.08161	-2.64799	-0.90982	-0.04446	0.79464	3.08663
ETH	-0.01341	2.14E-07	0.002	0.185	-3.11721	-0.63626	-0.04943	0.64698	2.80778
GAB	-0.019066	3.92E-04	0.005	0.1297	-4.6503	-0.7585	0.0266	1.0521	2.9602
GHA	-0.22088	8.64E-07	0.004	0.1891	-4.6036	-0.7854	0.0653	0.9259	4.4206
GIN	-0.040172	2.00E-16	0.003	0.5475	-3.4633	-0.7489	0.0282	0.8407	3.4562
GNQ	-0.024931	2.12E-09	0.004	0.2669	-4.8	-0.9298	0.0909	1.0086	3.8341
KEN	-0.014909	7.84E-06	0.003	0.1505	-2.5614	-0.9082	-0.0979	0.9024	4.3328
LBR	-0.050961	3.19E-12	0.006	0.3861	-5.8082	-1.2037	0.0361	1.3154	4.8078
LBY	-0.011042	5.01E-04	0.003	0.08674	-3.4071	-0.9486	-0.0678	0.9751	5.5876
LSO	-0.001181	1.18E-03	0.004	0.0006166	-4.1272	-1.4807	-0.0993	1.5032	4.2181
MAR	-0.01744	3.44E-09	0.003	0.1934	-4.4451	-1.2923	0.0284	1.2875	3.8972

MDG	-0.008485	3.89E-02	0.004	0.03191	-4.6279	-0.8901	-0.1081	1.0532	4.5956
MLI	-0.014778	1.21E-05	0.003	0.1515	-3.3191	-0.6955	-0.0572	0.8934	2.9524
MOZ	-0.009139	2.35E-02	0.004	0.04134	-4.0799	-1.0939	-0.0518	1.0867	4.6014
MRT	-0.020671	2.17E-08	0.003	0.245	-2.9844	-0.9335	0.0472	0.7782	2.7171
MWI	-0.018477	1.81E-03	0.006	0.08019	-5.6662	-1.2043	0.1717	1.2017	7.0808
NAM	-0.0006466	8.77E-01	0.004	0.0001893	-4.2969	-1.1374	-0.1989	0.6849	6.7976
NER	-0.007139	6.45E-02	0.004	0.0313	-3.4058	-0.7212	0.0868	0.7722	4.3451
NGA	-0.014961	5.12E-04	0.004	0.09844	-7.9324	-0.5473	0.1837	0.8922	3.6286
SDN	-0.032495	2.00E-16	0.003	0.4256	-2.7332	-0.9581	0.1599	0.9727	3.9191
SEN	-0.02812	2.00E-16	0.002	0.4758	-2.8209	-0.987	0.0366	0.6876	3.6725
SLE	-0.057589	2.00E-16	0.004	0.6542	-3.1287	-1.0012	-0.171	0.9413	3.7745
SLV	-0.030063	1.12E-05	0.007	0.1701	-8.0857	-1.6157	-0.0136	1.37	5.7554
SOM	0.01051	7.76E-04	0.003	0.1053	-2.33652	-0.61295	0.08567	0.58038	2.50439
SSD	-0.022848	5.11E-07	0.004	0.2057	-3.8764	-0.9331	0.1218	1.0791	2.9932
TCD	-0.014613	5.53E-04	0.004	0.1141	-2.9722	-0.9354	0.0408	0.8491	3.682
TGO	-0.017806	1.62E-05	0.004	0.1429	-4.9207	-0.9181	0.26437	1.0905	4.2196
TUN	0.008081	3.95E-02	0.004	0.03347	-3.6059	-1.0549	0.0231	0.9955	6.0856
TZA	0.005739	9.72E-02	0.003	0.02221	-4.6694	-1.0467	-0.0035	1.072	3.4672
UGA	-0.008039	4.09E-02	0.004	0.03648	-3.0538	-0.9776	-0.0226	0.8873	4.1223
ZAF	-0.007972	4.19E-04	0.002	0.07372	-2.6528	-1.0104	-0.2118	0.9701	3.9207
ZMB	-0.001333	7.63E-01	0.004	0.001035	-5.654	-0.9028	0.0596	0.8424	4.1035
ZWE	-0.007225	8.62E-02	0.004	0.02394	-4.0278	-1.1522	-0.2328	1.0939	4.3964

Table S4. Sensitivity analysis for possible projected drought changes. The selected six alternative drought scenarios are created to account for the full range of drought changes that could occur in the future, while all other covariate data will be kept at the 2016 level so that the effect of drought on cholera outbreak occurrence in this model can be isolated. Changes were only made up to the PDSI limits (+4 to -4) and if the scale limit was reached then this was taken as the value.

The six alternative drought scenarios for the sensitivity analysis are shown below:
Drought_average – Average national drought for 2000-2016
Drought_s1 – 2016 value + 0.5
Drought_s2 – 2016 value - 0.5
Drought_s3 – 2016 value + 1
Drought_s4 – 2016 value - 1
Drought_s5 – 2016 value + 2
Drought_s6 – 2016 value - 2

The table below shows the results for cholera outbreak occurrence predicted by the model using the different drought sensitivity scenarios, averaged to a continental level. This is shown at a national average below in the Supplementary Figure 6.

**Sensitivity analysis**	**Continental averages**
Drought_average	0.540106952
Drought_s1	0.561436986
Drought_s2	0.576812737
Drought_s3	0.553412242
Drought_s4	0.584072274
Drought_s5	0.53701047
Drought_s6	0.597395878

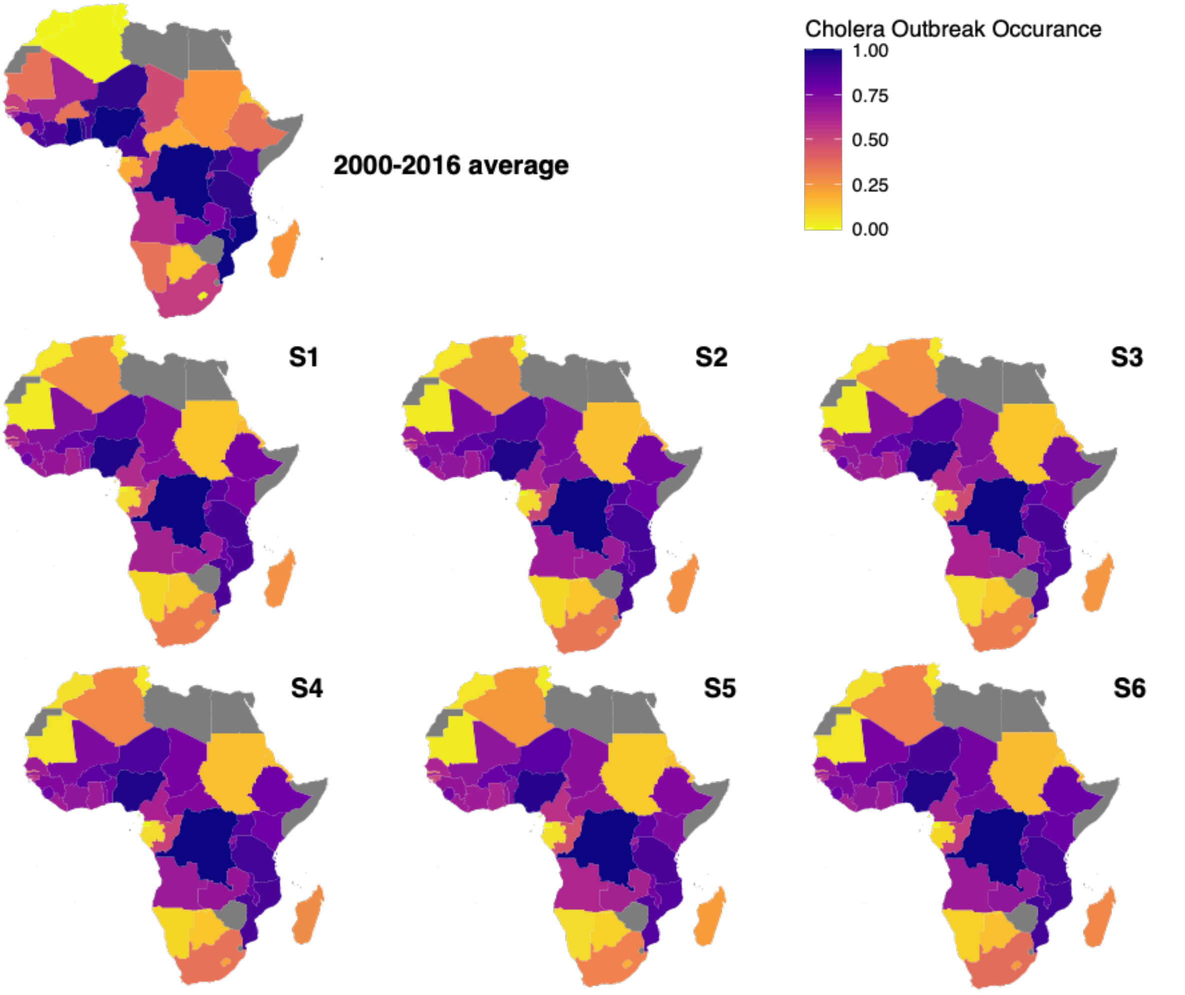

Fig. S7. Projected cholera outbreak occurrence using the best fit model fitted to the 2016 data and drought data altered in isolation from the 2000-2016 national averages as a baseline and the six different drought sensitivity analysis.

The map is our own work and the shapefiles are taken from: https://thematicmapping.org/downloads/world_borders.php, under CC-BY SA, allowing them to be shared and adapted.

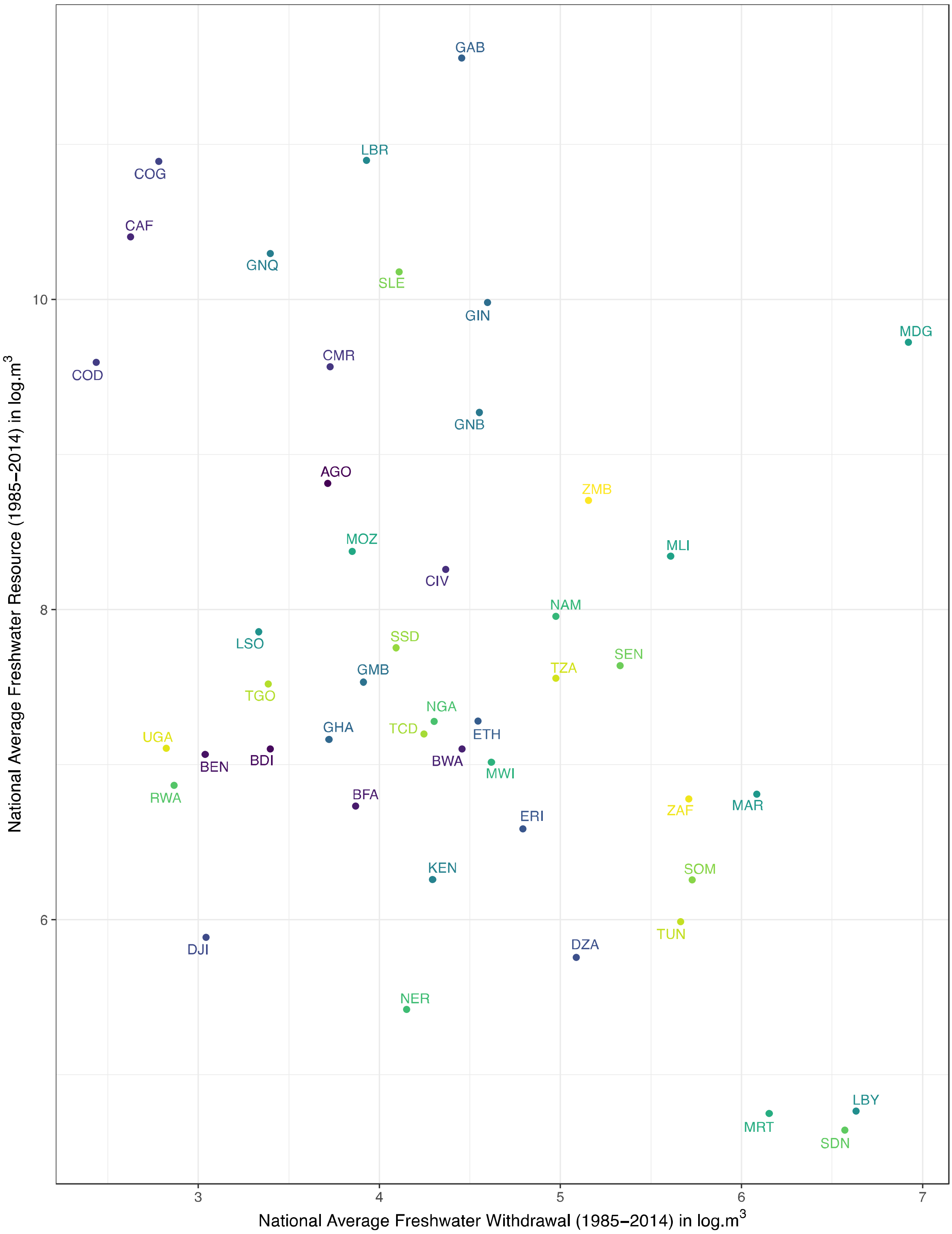

Fig. S8. Historical water security in Africa taken as freshwater withdrawal against freshwater resource. Both are taken as national per capita averages for the full dataset (1985-2014) in m³ and transformed on a logarithmic scale. Countries are identified using their alpha-3 codes and the data is taken from: Ritchie, H., 2017. Water Use and Stress. [On-line]. Our World In Data. Available from: https://ourworldindata.org/water-use-stress (accessed 26 October 2020).

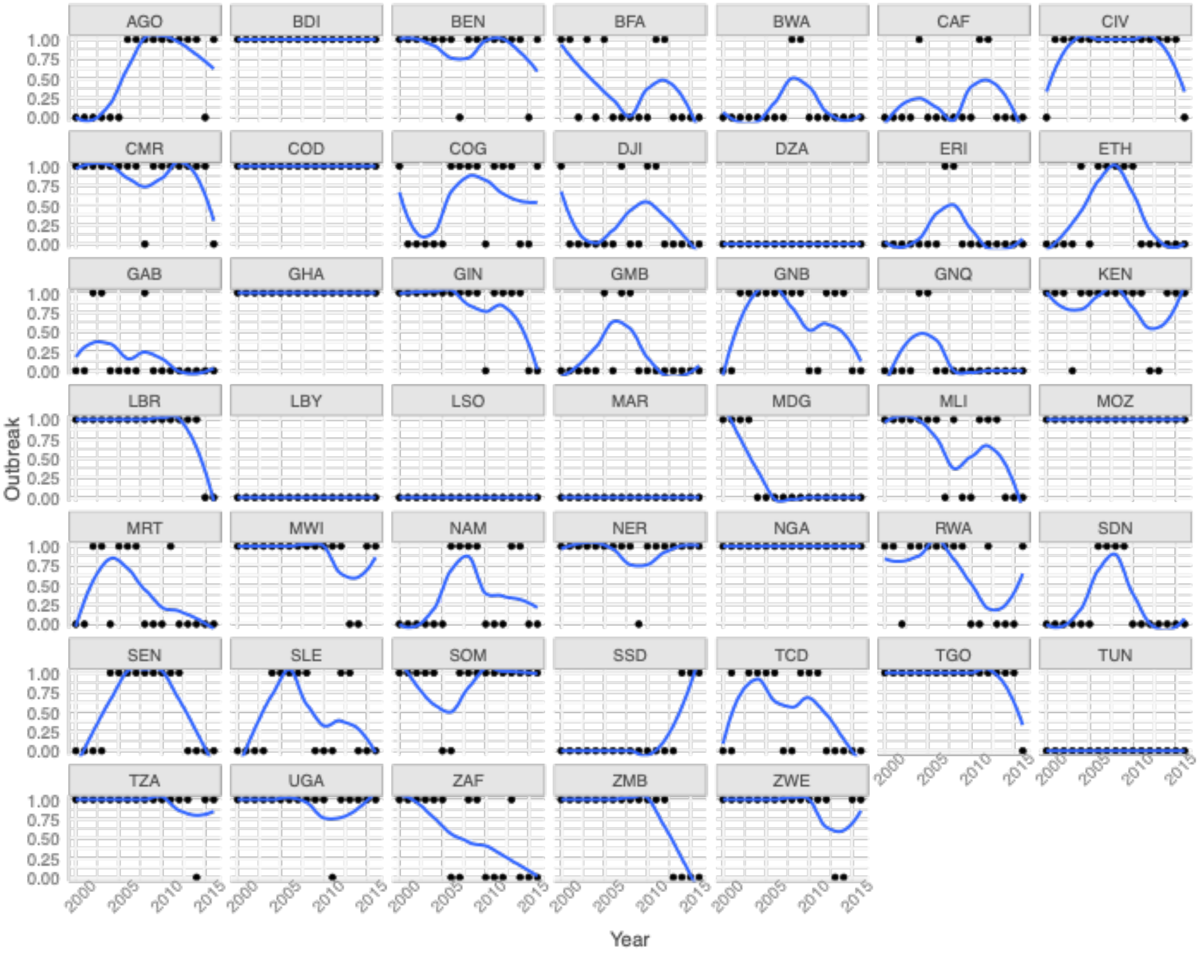

Fig. S9. Loess curves showing year and outbreak for each country (2000-2016) with a smoothed trend line and standard error.

Information 4. Diagnostic results for temporal and spatial effect testing.

Time series for lm(Outbreak ~ Year), showing year as significant at p=<0.05

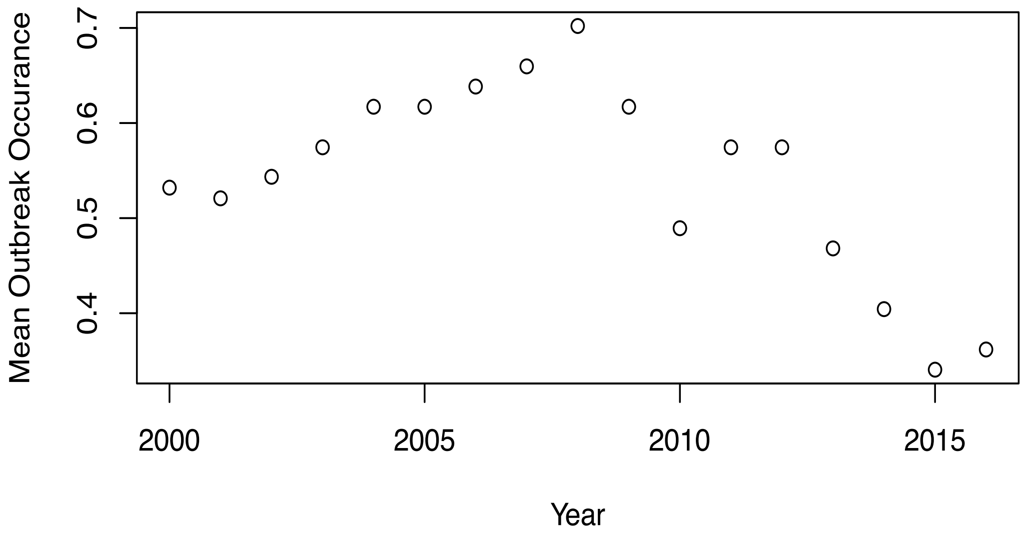

Model performance for lm(Outbreak ~ Year) shows some temporal autocorrelation

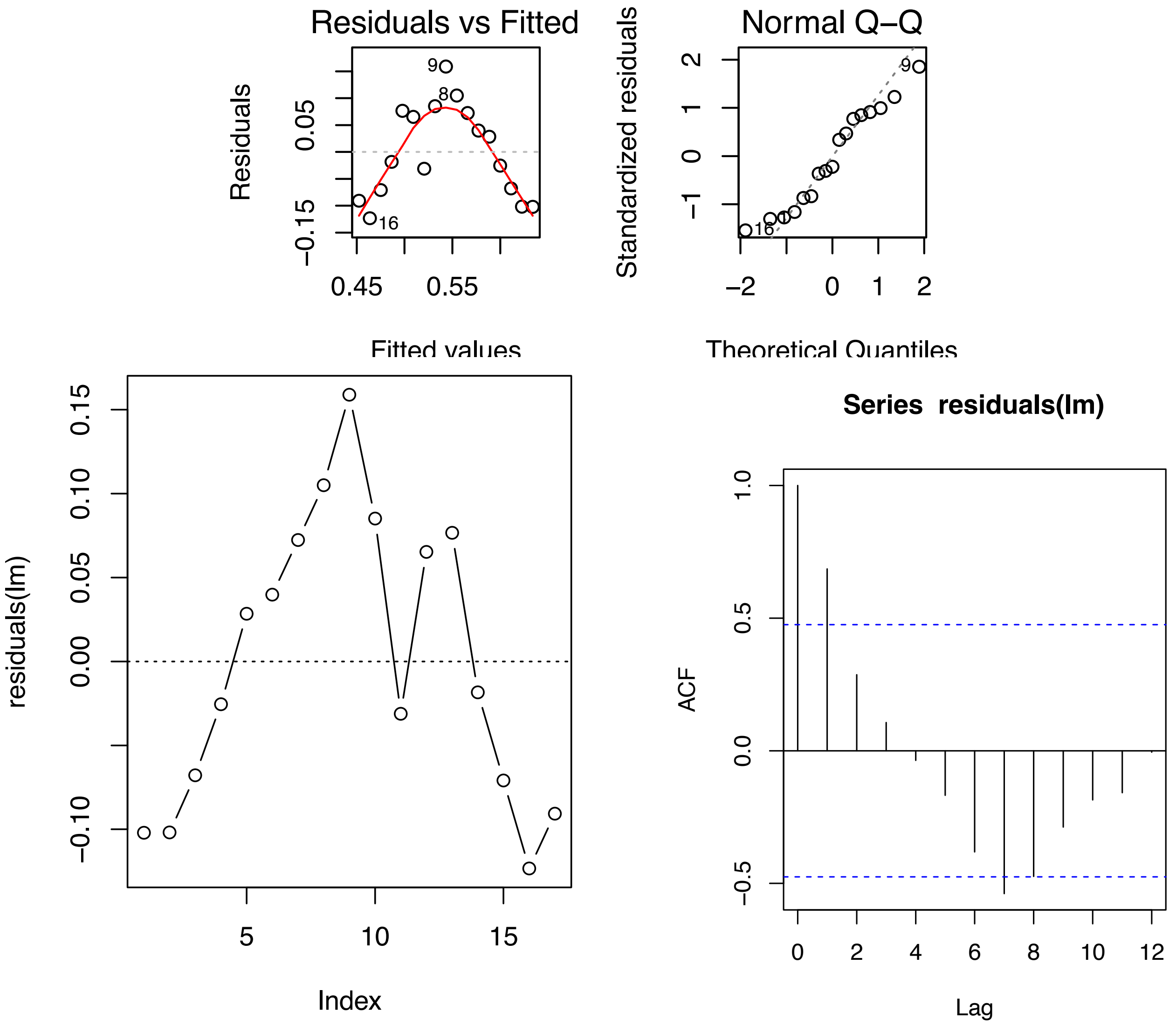

Re-estimate the linear trend and model performance account for autocorrelation
gls(Outbreak~ Year, correlation = corAR1(form = ~Year))

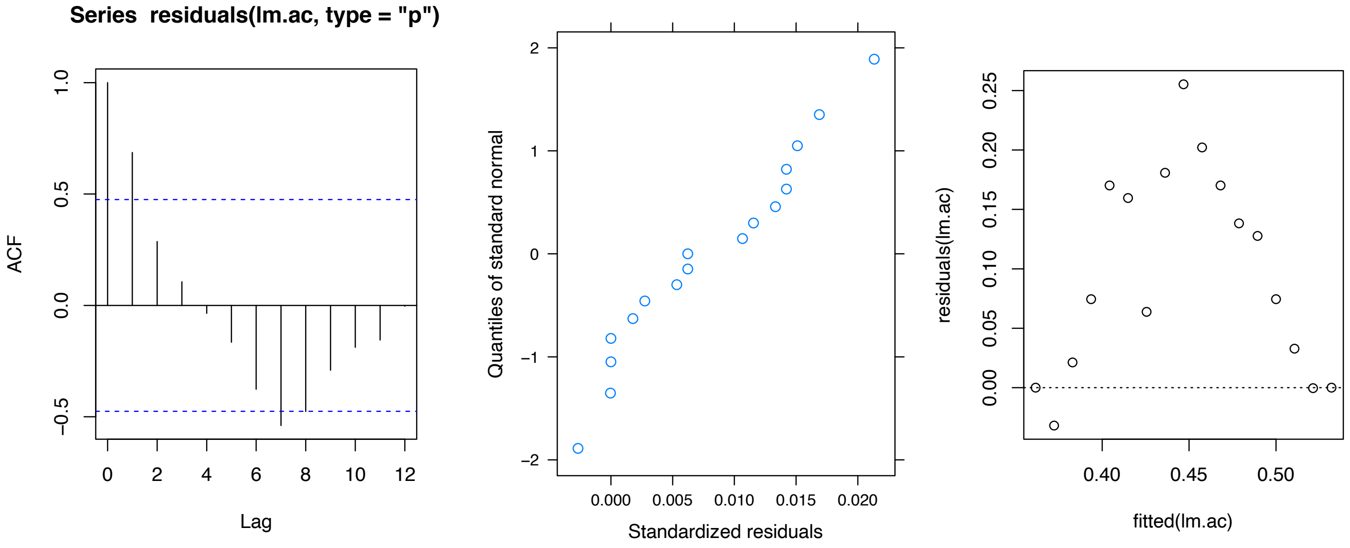

Year is no longer significant, but the intercept remains the same
When the two linear models are compared, with or without correction for autocorrelation, there is no appreciable difference, with a difference of 1 for the AIC.
The two multivariate glm, selected by the covariate selection process were then run through LOO to assess for an appreciable model performance difference.
Model accuracy was different by 0.02 (0.7580214 to 0.7755418) and cross validation prediction error also by 0.02 (0.1827365 to 0.1653325).
R packages used include: nlme, MuMIn, car, carat, boot.
